# Two ways to make your robot proactive: Reasoning about human intentions or reasoning about possible futures

**DOI:** 10.3389/frobt.2022.929267

**Published:** 2022-08-15

**Authors:** Sera Buyukgoz, Jasmin Grosinger, Mohamed Chetouani, Alessandro Saffiotti

**Affiliations:** ^1^ SoftBank Robotics Europe, Paris, France; ^2^ Sorbonne University, Institute for Intelligent Systems and Robotics, CNRS UMR 7222, Paris, France; ^3^ AASS Cognitive Robotic Systems Lab School of Science and Technology, Orebro University, Orebro, Sweden

**Keywords:** proactive agents, human intentions, autonomous robots, social robot, human-centered AI, human–robot interaction

## Abstract

Robots sharing their space with humans need to be proactive to be helpful. Proactive robots can act on their own initiatives in an anticipatory way to benefit humans. In this work, we investigate two ways to make robots proactive. One way is to recognize human intentions and to act to fulfill them, like opening the door that you are about to cross. The other way is to reason about possible future threats or opportunities and to act to prevent or to foster them, like recommending you to take an umbrella since rain has been forecast. In this article, we present approaches to realize these two types of proactive behavior. We then present an integrated system that can generate proactive robot behavior by reasoning on both factors: intentions and predictions. We illustrate our system on a sample use case including a domestic robot and a human. We first run this use case with the two separate proactive systems, intention-based and prediction-based, and then run it with our integrated system. The results show that the integrated system is able to consider a broader variety of aspects that are required for proactivity.

## 1 Introduction

Humans can act on their own initiative. Imagine the following scenario: you see your flatmate preparing to leave for a hiking trip in a rainy zone. It is quite likely that you will give your flatmate some advice like checking the weather forecast or taking some extra equipment. Such behavior even occurs between strangers. When people see a person holding garbage and looking around, they tend to show where the garbage bin is since they recognized the person’s intention to dispose of their garbage. This type of intuitive interaction is common among humans, and it is already observed in infants (Warneken and Tomasello, 2006). The question is, what happens if one of the actors is a robot? The robot should be able to recognize and reason about the human’s intentions; to reason about the current and forecasted states of the environment; to understand what states may be preferable to others; and to foresee problems that the human could face. The robot should also be able to reason about the potential effects of its own actions and select and perform actions that support the human given this context. The behavior of initiating own action taking into account all these aspects is called *proactive behavior*.

Most of the existing work on human–robot interactions rely on the human taking the initiative: the human sets a request, and the robot generates and executes a plan to satisfy it. However, in the above examples of human-to-human interaction, there is no explicit given goal. The interaction works because humans are able to assess other humans’ intentions, anticipate consequences, and reason about preferred states. In this article, we discuss proactive human–robot interaction, that is, interactions where the robot behaves by acting on its own initiative, in an anticipatory way, and without being given an explicit goal (Grant and Ashford, 2008; Grosinger et al., 2019; Peng et al., 2019). We consider two types of proactive robot behavior: one in which the robot understands the human’s intentions and helps the human to achieve them and the other in which the robot foresees possible future situations that are undesirable (or desirable) according to the human’s preferences and acts to avoid (or foster) them.

In particular, we propose a framework that identifies opportunities for acting and selects some of them for execution. *Opportunities* here are formal concepts grounded in the relationship among actions, preferences, and state predictions. Our framework includes two main mechanisms that contribute to initiating the proactive behavior—*human intention recognition and reasoning* (*HIRR*) and *equilibrium maintenance* (*EqM*). The former mechanism is based on recognizing human intent from a known list of possible intents that the human can have. The latter one is based on predicting how the state may evolve in time and comparing preferences of states resulting from different actions (or inaction) (Grosinger et al., 2019). These two mechanisms correspond to the two types of proactive behavior mentioned above: intention-based and prediction-based. The whole framework includes provisions to combine these two mechanisms into an integrated proactive system. (For a deeper discussion on different types of proactivity, see Grosinger (2022).)

The general aim of this article is to shed some light on the notion of proactivity and on its use for service robots interacting with humans. We focus in particular on the difference between the two different types of proactivity mentioned above, which are often found (but not distinguished) in the literature, and we show that a combined solution is possible and perhaps needed. Within this aim, the more specific contributions of this article are as follows: 1) we propose a novel method based on human intention recognition to generate intention-based proactive robot behavior, which we call (*HIRR*); 2) we adapt an existing method based on temporal predictions and state preferences, called (*EqM*), to generate prediction-based proactive robot behavior; 3) we define an architecture to combine both methods to create a proactive robot that considers both human intentions and temporal predictions; and 4) we compare all these using a sample case study.

The rest of this article is organized as follows. The next section presents the necessary background together with related work on intention recognition, proactivity, and their combination. In Section 3, we define our systems for intention-based proactivity and for prediction-based proactivity, together with their integration. Section 4 describes the implementation and shows the results of a task involving a simulated domestic robot and a human. At last, in Section 5, we discuss our results and conclude.

## 2 Background and related work

In our work, we combine intention recognition and temporal predictions to generate proactive behavior. Here we provide the relevant background and related work on these research areas.

### 2.1 Intention recognition

To assist humans, a robot requires some knowledge of the human’s goals and intentions. In belief-desire-intention (BDI) models (Rao and Georgeff, 1995), the agent represents the environment in terms of beliefs that are true. A set of desires, representing the agent’s goals, guides the agent’s behavior. We may or may not know the agent’s goals. The intention represents the path that the agent is currently taking to reach a goal. Bratman (1989) points out that the concept of intention is used to characterize both the human’s actions and mind (mental states). Actions are considered as done with a certain intention. Humans attribute mental states of intending to other agents such as having an intention to act in certain ways now or later. In this article, we consider an intention to be a mental state that is expressed through goal-directed actions.

*Intention recognition* is the process of inferring an agent’s intention by analyzing his or her actions and the actions’ effects on the environment (Han and Pereira, 2013). Approaches in action recognition, goal recognition, and plan recognition have been used to infer intention. According to Van-Horenbeke and Peer (2021), intention recognition systems can be classified as logic-based, classical machine learning, deep learning, and brain-inspired approaches, or they can be classified in terms of the behavior of the observed human toward the observer. We take here a simplified view and consider two classes of intention recognition approaches: logic-based and probabilistic. Logic-based approaches are defined by a set of domain-independent rules that capture the relevant knowledge to infer the human’s intention through deduction (Sukthankar et al., 2014). The knowledge can be represented in different ways, including using plan representation languages like STRIPS and PDDL that describe the state of the environment and the effects of the possible actions. Logic-based approaches work well in highly structured environments. Logic representation can define different kinds of relationships depending on the problem. These relationships allow us to recognize humans’ intentions based on observations. Another advantage of logic-based approaches is that they are highly expressive. The reasoning result can potentially be traceable and human-understandable. However, many logic-based approaches assume that human is rational and try to find the optimal intention that best fits the observations, while humans often act in nonrational ways (Dreyfus, 2007). This makes logic-based approaches less reliable in real-world problems. The uncertainty in humans’ rationality might be addressed by a combination of logic-based with probabilistic reasoning techniques. Probabilistic approaches exploit Bayesian networks and Markov models. Bayesian networks are generative probabilistic graphical models that represent random variables as nodes and conditional dependencies as arrows between them (Van-Horenbeke and Peer, 2021). They can provide the probability distribution of any set of random variables given another set of observed variables. Some planning systems use Bayesian inference to reason about intention. Such approaches are referred to as Bayesian inverse planning. Ramírez and Geffner (2009) propose an approximate planning method that generates a set of possible goals by using Bayesian inverse planning methods on classical planning. The method assumes that humans are perfectly rational, which means they only optimally pursue their goals. As a result of this, the indecisive behavior of humans is not tolerated. This limitation is partly addressed by Ramírez and Geffner (2010), who introduced a more general formulation. Persiani (2020) offers an example of using Bayesian inference in a logic-based approach. The authors use classical planning to generate an action plan for each goal, and then they use a Bayesian prior function to infer human intention. Probabilistic approaches are able to handle uncertainty and can therefore handle real-world settings such as nonrational agents, interrupted plans, and partial observability (Van-Horenbeke and Peer, 2021). On the other hand, they are less expressive than logic-based systems, since it is hard to understand the reasoning behind the result. Scalability is another well-known difficulty with probabilistic approaches.

In this work, we adopt a logic-based approach as performed by Persiani (2020): we represent the robot’s knowledge in a symbolic form, which the robot uses to plan its actions. We assume rational humans. By this, we mean humans follow the *principle of rationality* defined by Newell (1982): “If an agent has knowledge that one of its actions will lead to one of its goals, then the agent will select this action.” The approach gives us the advantage of getting results that are easily traceable and human-readable.

### 2.2 Proactivity

*Proactive* AI systems and robots are opposed to *reactive* AI systems, which respond to explicit requests or external events. In organizational psychology, proactive behavior is understood as an *anticipatory self-initiated action* (Grant and Ashford, 2008). When it comes to artificial agents, though, we lack a clear definition of proactivity. Drawing inspiration from the human proactive process, we can identify the functionalities that are needed for artificial proactivity: context-awareness, activity recognition, goal reasoning, planning, plan execution, and execution monitoring. Each one of these functionalities in itself has been the subject of active research (Beetz et al., 2016; Ghallab et al., 2016; Aha, 2018; Wang et al., 2019; Doush et al., 2020). Proactivity needs to contemplate these areas jointly and in a separate process to each. Context-awareness is not the central topic in proactivity, but it is used to understand what the current situation is, and with this knowledge, it is possible to decide how to act. Goal reasoning deals with questions about generating, selecting, maintaining, and dispatching goals for execution (Aha, 2018). Planning can be described as searching and selecting an optimal action trajectory to a goal that is given externally by a human or by some trigger. Proactivity resides on the abstraction level above. It is finding the acting decisions or goals that should be planned for; hence, it produces the input to a planner. In conclusion, plan execution and monitoring are employed by proactivity to enact the acting decision inferred and to invoke new reasoning on proactivity when execution fails.

In recent times, there has been a number of promising works in the field of artificial proactivity. Baraglia et al. (2017) address the question of whether and when a robot should take initiative during joint human–robot task execution. The domain used is table-top manipulation tasks. Baraglia et al. (2017) used dynamic Bayesian networks to predict environmental states and the robot’s actions to reach them. Initiation of action is based on a hard trigger that at least one executable action exists that does not conflict with human actions. In contrast, in the work presented in this article, we aim to find a general solution where acting is based on reasoning on first principles, rather than on hard-coded triggers or rules. Bremner et al. (2019) present a control architecture based on the BDI model incorporating an extra ethical layer to achieve agents that are proactive, transparent, ethical, and verifiable. They do anticipation through embedded simulation of the robot and other agents. Thereby, the robot can test what-if-hypotheses, e.g., what if I carry out action *x*? The robot controller is given a set of goals, tasks, and actions and thereof generates behavior alternatives, i.e., plans. The simulation module simulates them and predicts their outcome. The ethical layer evaluates the plans and if needed invokes the planner module to find new plans proactively. Note that proactive plans are only generated if previously generated plans from given goals fail against some given ethical rules, which admittedly limits generality. The approach that we propose below is more general since it generates proactive actions from the first principles. On the other hand, our approach does not take ethics into account. Umbrico et al. (2020a and b) present a general-purpose cognitive architecture with the aim to realize a socially assistive robot (SAR), specifically, for supporting elderly people in their homes. Their highly integrated framework includes a robot, a heterogeneous environment, and physiological sensors and can do state assessments using these sensors and an extensive ontology. However, their approach to making the SAR proactive is based on hard-wired rules like “user need: high blood pressure → robot action: blood pressure monitoring in context *sleeping*.” Peng et al. (2019) are mainly interested in finding the right level of proactivity. They use hand-coded policies for guiding the behavior of a robotic shopping assistant and find that users prefer medium proactivity over high or low proactivity.

#### 2.2.1 Equilibrium maintenance

In this work, we use *EqM*, a mechanism proposed by Grosinger et al. (2019) for achieving proactivity. *EqM* autonomously infers acting decisions based on a temporal prediction of one or several steps. *EqM* is a general approach based on a formal model and thereby affords domain independence. This model is modular and can cope with different agent capabilities, different preferences, or different predictive models. The relationship between situations and triggered actions is not hard coded: decisions are inferred at run time by coupling action with state, predicted states and preferences, and choosing among acting alternatives at run time. In Section 3.2, we give a deeper description of *EqM*.

A work that has comparable ideas to the ones in *EqM* is the one on *α*POMDPs by Martins et al. (2019). With the aim to develop a technique for user-adaptive decision-making in social robots, they extend the classical POMDP formulation so that it simultaneously maintains the user in valuable states and encourages the robot to explore new states for learning the impact of its actions on the user. As in all flavors of (PO)MDPs, however, the overall objective is to find an optimal, reward-maximizing policy for action selection; by contrast, the aim of *EqM* is to maintain an overall desirable world state, be it by acting or by inaction. Instead of rewarding actions, as done in MDPs, *EqM* evaluates the achieved effects of the actions (or of being inactive).

*EqM* is also reminiscent of supervisory control of discrete event systems (Skulimowski, 1990), especially when anticipatory feedback is used (Skulimowski, 2016). Different from these, however, *EqM* does fast local reasoning, and it does not address the computationally challenging problem of computing an optimal global policy.

### 2.3 From intention recognition to proactivity

Several authors have proposed reactive systems based on intention recognition. Zhang et al. (2015) provide a framework for general proactive support in human–robot teaming based on task decomposition, where the priorities of subtasks depend on the current situation. The robot reprioritizes its own goals to support humans according to recognized intentions. Intentions are recognized by Bayesian inference following Ramírez and Geffner (2010). Each goal’s probability depends on the agent’s past and/or current belief, and the goal with the highest probability from a candidate goal set is recognized as the current intention. Our framework is similar to that of Zhang et al. (2015) in linking intention recognition with the proactive behavior of the robot. In our case, however, the robot does not have its own independent tasks to achieve: the robot’s only objective is to help the human proactively by enacting actions to reach his or her goal.

Sirithunge et al. (2019) provide a review of proactivity focused on perception: robots perceive the situation and user intention by human body language before approaching the human. The review aims to identify cues and techniques to evaluate the suitability of proactive interaction. Their idea of proactivity is that the robot identifies a requirement by the human and acts immediately. This differs from our understanding of reasoning on proactivity: we generate proactive agent behavior by considering the overall environment, the human’s intentions, the overall preferences, and the prediction on how the state will evolve. This can result in the agent acting now or later or not at all.

Harman and Simoens (2020) aimed at predicting what action a human is likely to perform next, based on previous actions observed through pervasive sensors in a smart environment. Predictions can enable a robot to proactively assist humans by autonomously executing an action on their behalf. The so-called *action graphs* are introduced to model order constraints between actions. The program flow is as follows: 1) action by the human is observed; 2) next actions are predicted; 3) predicted actions are mapped to a goal state; 4) a plan for the robot and a plan for the human are created to reach the goal state; and 5) the robot decides which action it should perform by comparing the robot’s and the human’s plan. The work presented in this article shares some traits with the one by Harman and Simoens (2020): in both cases, we reason on human intentions and make predictions about future states using action models. In our case, however, predictions are made on how the system evolves *with* and *without* robot actions, and proactive actions are taken by comparing those predictions. In particular, in our case, these actions might *not* be part of the human’s plan. In conclusion, the trigger to perform proactivity reasoning in the study of Harman and Simoens (2020) is human action, while in our work, this trigger is any state change, be it caused by human action or by the environment.

In the work of Liu et al. (2021), the authors’ aim is to recognize and learn human intentions online and provide robot assistance proactively in a collaborative assembly task. They introduced the evolving hidden Markov model which is a probabilistic model to unify human intention inference and incremental human intention learning in real time. Liu et al. (2021) conducted experiments where a fixed robot arm assists a human according to recognized intention in assembling cubes marked by a fiducial mark on top. One such configuration corresponds to one particular intention. The human starts to assemble the cubes and the robot proactively finishes the shape as soon as the intention is recognized or when a maximally probable intention is found by doing a one-step prediction. In the study of Liu et al. (2021), proactivity results from strict one-to-one links where one recognized intention always leads to the same action sequence, using a one-step prediction. In our approach, proactive robot behavior too can be based on recognizing intentions and their corresponding action sequences, but it can also be inferred from first principles at run time using multiple steps prediction.

## 3 System

**FIGURE 1:**
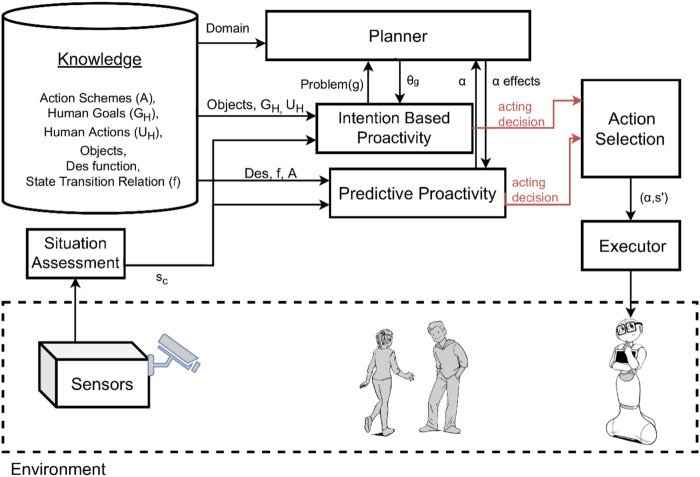
System model: an autonomous system that initiates proactive behavior according to the situation of the environment, including the human.

We claimed that to initiate proactive behavior, robots must be equipped with the abilities to recognize human intentions, to predict possible future states and reason about their desirability, and to generate and enact opportunities for actions that can lead to more desirable states. To combine these abilities, we propose the general system model shown in Figure 1.

The system includes different components to offer a fully autonomous interaction, namely, a situation assessment, a knowledge component, a planner, an intention-based proactivity component, a predictive proactivity component, an action selection component, and lastly an executor. The **situation assessment** and the **executor** components act as interfaces to the physical environment. They respectively collect and induce changes from/to the environment. The **knowledge** component represents a model of the environment. This model encodes the state evolution of the world, the set of goals of a human, action plans of how the human can reach his or her goals, robot capabilities as a set of action schemes, the state transition relation, and a desirability function to compute the degree of desirability of a state.

To be more specific, we model the environment and its dynamics using a standard dynamic system Σ = ⟨*S*, *U*, *f*⟩ where *S* is the set of states, *U* is the finite set of external inputs (robot actions or human actions), and *f* ⊆ *S* × *U* × *S* is the transition relation. The system’s dynamics is modeled by the relation *f*(*s*, *u*, *s*′), which holds if Σ can go from *s* to *s*′ when input *u* is applied in *s*. To give *S* a structure, we rely on a symbolic representation of world states. Given a finite set L of predicates, we let S⊆P(L) and denote the current state by *s*_*c*_. Each state *s* ∈ *S* is thereby completely determined by the predicates that are true in *s*. We denote the set of human goals by *G*_*H*_ ⊆ *S*. Each goal *g* ∈ *G*_*H*_ is determined by predicates that are true in *g*. Given a goal *g*, we denote any state in *S* where all predicates in *g* (and potentially more) are true by *s*_*g*_, hence *g* ⊆ *s*_*g*_. At last, we denote the set of all states *s*_*g*_ by *S*_*g*_ ⊆ *S*, where the predicates of *g* are true.

The **planner** is an off-the-shelf planner able to create a sequence of actions that leads from the current state to a goal state. In our implementation, we use *Fastdownward* ^1^, a domain-independent planner based on PDDL, the planning domain definition language (Ghallab et al., 1998). Both the human’s plans and the robot’s plans are formulated in PDDL, a standard language to define planning domains and problems. The planning *domain* includes the predicates of L used for describing states and operators that model the available actions of humans and robots. The planning *problem* includes information about the available objects, the current state *s*_*c*_, and the goal of the human *g* ∈ *G*_*H*_. Given a domain and a problem, the planner finds the shortest plan *θ*_*g*_(*s*) between the current state *s*_*c*_ and the given goal *g*. This plan represents the sequence of actions that the agent should do to reach any state *s*_*g*_ where all predicates of *g* are true.

The **intention-based proactivity** component and the **predictive proactivity** component are both able to generate proactive behavior, but they use two different methods which we describe below. At last, the **action selection** component integrates the decisions generated by those two methods into an overall proactive behavior to be executed by the robot.

To describe the main contribution of this article, i.e., the integration of intention-based and predictive proactivity, we first need to introduce individual systems which it is based on. In Section 3.1, we present our novel approach for intention-based proactivity, *HIRR*. In Section 3.2, we recall our existing approach to *EqM*, and in Section 3.3, we describe the integration of *HIRR* and *EqM*.

### 3.1 Intention-based proactivity: Human intention recognition and reasoning

Experimental psychology shows that humans can interpret others’ intentions by observing their actions, which is part of the so-called theory of mind (Premack and Woodruff, 1978). Interpreting actions in terms of their final goal may give hints on why a human performed those actions and hence make us able to infer those human intentions (Han and Pereira, 2013). Inspired by these concepts, we define a framework called *HIRR* for generating proactive behavior based on intention recognition. Intention recognition applies *inverse planning* ^2^ rules to recognize the intentions of a human in the form of an action plan. A robot can then *proactively* enact the next action in that action plan on behalf of the human, or it can inform the human on which action to take next to reach their goal.

There are different methods to recognize human intentions. We select inverse planning since this is a straight-through logic-based approach for fully observable systems. The approach has been widely used in other systems for intention recognition (Han and Pereira, 2013; Farrell and Ware, 2020; Persiani, 2020). While planning synthesizes a sequence of actions to reach a goal, in inverse planning we observe the execution of a sequence of actions to infer the human’s goal and the corresponding plan. The assumption of full observability may reduce the robustness of plan recognition in real settings, since missing to observe an action might lead to a plan not being recognized. On the other hand, goal inference has some degree of robustness with respect to variations in the sequence in which human actions are observed, that is, different observed sequences used to reach the same goal can be recognized as the same intention. Different sequences may arise because of asynchronous sensors or because the human performs the actions in a different order. This invariance property allows our system to tolerate some amount of uncertainty.

Once the user has committed to reaching a goal, we say the user *intends* to reach that goal *g*. We define an intention i(s) in state *s* to be an action plan *θ*_*g*_(*s*) to reach goal *g* from state *s*. We infer human intentions I(s) as defined in Eq. 1:

$$I\left(s\right)=\left\{\theta_{\hat{g}}\left(s\right)|\hat{g}\in \underset{g\in G_{H}}{\mathrm{argmin}}\left(len\left(\theta_{g}\left(s\right)\right)\right)\right\}$$ (1)

In words, for each goal *g* in the set *G*_*H*_ of a human’s potential goals, we use our planner to compute the shortest plan *θ*_*g*_(*s*) that the human can perform to reach *g* from the current state *s*. We then select the goal $\hat{g}$ in *G*_*H*_ to which the shortest of these plans leads: $\theta_{\hat{g}}\left(s\right)$ . The rationale behind this is that $\theta_{\hat{g}}\left(s\right)$ has the shortest number of actions left to be executed; that is, the human already has executed a large part of this plan. Since we assume that the human is rational, it is plausible to infer that the human intends to do all the remaining actions in $\theta_{\hat{g}}\left(s\right)$ to reach $\hat{g}$ from *s*. Therefore, we take the action list in $\theta_{\hat{g}}\left(s\right)$ to be the intention i(s) of the human in state *s*. This strategy has been originally proposed in logic-based approaches by Persiani (2020).

Equation 1 is implemented by Algorithm 1 called *HIRR* that returns the intention i(s)∈I(s) . The returned intention is the residual action plan $\theta_{\hat{g}}\left(s\right)$ of the human’s recognized intention to be enacted proactively by the robot. If the cardinality of the set of goals with the shortest residual action plans, i.e., the cardinality of the set of intentions, is not 1, the intention is not recognized or it is ambiguous and an empty set is returned.

Algorithm 1 *HIRR*(*s*, *G*_*H*_)

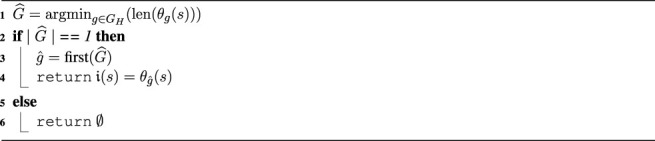

### 3.2 Predictive proactivity: Equilibrium maintenance

For doing reasoning on predictive proactivity we use a computational framework called *EqM*, described in detail by Grosinger et al. (2019). We only give a brief overview of the framework here, the interested reader is referred to the cited reference for details.

In our framework, the evolution of system Σ by itself, that is, when no robot action is performed, is modeled by its *free-run behavior* *F* ^*k*^. *F* ^*k*^ determines the set of states that can be reached from an initial state *s* in *k* steps when applying the null input ⊥.

$$\begin{array}{ll} F^{0}\left(s\right) & =\left\{s\right\}\\ F^{k}\left(s\right) & =\left\{s^{\prime }\in S|\exists s^{\prime \prime }:f\left(s,⊥,s^{\prime \prime }\right)\wedge s^{\prime }\in F^{k-1}\left(s^{\prime \prime }\right)\right\}. \end{array}$$

Desirable and undesirable states are modeled by *Des*, a fuzzy set of *S*. The membership function *μ*_*Des*_: *S* → [0, 1] measures the degree by which a state *s* is desirable. *Des* is extended from states to sets of states in an obvious way: *μ*_*Des*_(*X*) = inf_*s*∈*X*_(*μ*_*Des*_(*s*)), where *X* ⊆ *S*. We abbreviate *μ*_*Des*_(⋅) as *Des*(⋅).

The available robot actions are modeled by *action schemes*: Partial functions $\alpha :P\left(S\right)\to P^{+}\left(S\right)$ that describe how states can be transformed into other states by robot acting. An action scheme *α* abstracts all details of action: *α*(*X*) = *Y* only says that there is a way to go from any state in the set of states *X* to some state in set *Y*. Action schemes can be at any level of abstraction, from simple actions that can be executed directly to sequential action plans, or policies, or high-level goals for one or multiple planners. Applying an action scheme *α* in a state *s* may bring about effects that are (or are not) desirable, possibly in *k* steps in the future. We call the degree to which an applied action scheme achieves desirable effects *benefit*:

$$\mathrm{Bnf}\left(\alpha ,s,k\right)=\underset{X\in \mathrm{dom}\left(\alpha ,s\right)}{\inf}\mathrm{Des}\left(F^{k}\left(\alpha \left(X\right)\right)\right),$$ (2)

where *F* ^*k*^(*X*) = ⋃_*s*∈*X*_ *F* ^*k*^(*s*) and *dom*(*α*, *s*) is the domain of *α* relevant in *s*.

With this background, Grosinger et al. (2019) define seven different types of *opportunity* for acting, which are the foundation of proactivity by *EqM*. We write *Opp*_*i*_(*α*, *s*, *k*) to mean that applying action scheme *α* in state *s* is an opportunity of type *i*, by looking *k* steps into the future.

$$\begin{array}{ll} {\mathrm{Opp}}_{0}\left(\alpha ,s,0\right) & =\min\left(1-\mathrm{Des}\left(s\right),\mathrm{Bnf}\left(\alpha ,s\right)\right)\\ {\mathrm{Opp}}_{1}\left(\alpha ,s,k\right) & =\min\left(1-\mathrm{Des}\left(s\right),\underset{s^{\prime }\in F^{k}\left(s\right)}{\sup}\left(\mathrm{Bnf}\left(\alpha ,s^{\prime }\right)\right)\right)\\ {\mathrm{Opp}}_{2}\left(\alpha ,s,k\right) & =\min\left(1-\mathrm{Des}\left(s\right),\underset{s^{\prime }\in F^{k}\left(s\right)}{\inf}\left(\mathrm{Bnf}\left(\alpha ,s^{\prime }\right)\right)\right)\\ {\mathrm{Opp}}_{3}\left(\alpha ,s,k\right) & =\underset{s^{\prime }\in F^{k}\left(s\right)}{\sup}\left(\min\left(1-\mathrm{Des}\left(s^{\prime }\right),\mathrm{Bnf}\left(\alpha ,s^{\prime }\right)\right)\right)\\ {\mathrm{Opp}}_{4}\left(\alpha ,s,k\right) & =\underset{s^{\prime }\in F^{k}\left(s\right)}{\inf}\left(\min\left(1-\mathrm{Des}\left(s^{\prime }\right),\mathrm{Bnf}\left(\alpha ,s^{\prime }\right)\right)\right)\\ {\mathrm{Opp}}_{5}\left(\alpha ,s,k\right) & =\min\left(\underset{s^{\prime }\in F^{k}\left(s\right)}{\sup}\left(1-\mathrm{Des}\left(s^{\prime }\right)\right),\mathrm{Bnf}\left(\alpha ,s,k\right)\right)\\ {\mathrm{Opp}}_{6}\left(\alpha ,s,k\right) & =\min\left(\underset{s^{\prime }\in F^{k}\left(s\right)}{\inf}\left(1-\mathrm{Des}\left(s^{\prime }\right)\right),\mathrm{Bnf}\left(\alpha ,s,k\right)\right) \end{array}$$

To understand these opportunity types, consider for example the first type *Opp*_0_: the degree by which *α* is an opportunity of type 0 is the minimum of (i) the degree by which the current state *s* is undesirable and (ii) the benefit of acting now. In an intuitive manner, *α* is an opportunity of type 0 if (and to the extent) we are in an undesirable state, but enacting *α* would bring us to a desirable one. As another example, consider *Opp*_5_: here, we compute the minimum of (i) the maximum undesirability of future states and (ii) the future benefit of acting now: intuitively, *α* is an opportunity of type 5 if (and to the extent) some future states within a look-ahead *k* are undesirable, but if we enact *α* now, then all the *k*-step future states will be desirable. For a detailed explanation of the rest of the opportunity types, see Grosinger et al. (2019).

At last, we can define what it means for a system to be in equilibrium from a proactivity perspective.

$$\mathrm{Eq}\left(s,K\right)=1-\underset{k,i,\alpha }{\sup}{\mathrm{Opp}}_{i}\left(\alpha ,s,k\right),$$ (3)

where *k* ∈ [0, *K*], *i* ∈ [0, 6], and *α* ∈ *A*, where *A* is the set of all action schemes. In an intuitive manner, equilibrium is a measure of lack of opportunities: if there are big opportunities, then the system is very much out of equilibrium; if there are small opportunities, then the system is close to being in equilibrium; if there are no opportunities at all, then the system is fully in equilibrium. The notion of equilibrium is used in the *EqM* algorithm to achieve agent proactivity, as shown in Algorithm 2 ^3^.

Algorithm 2 *EqM*(*s*, *K*)

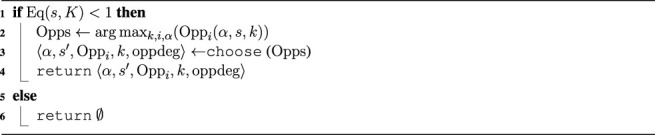

### 3.3 Action selection: Integrating human intention recognition and reasoning and equilibrium maintenance

*HIRR* and *EqM* are complementary approaches that create proactive acting in different ways. We now explore how to integrate the two systems. The action selection component in Figure 1 integrates the approaches at the result phase after each system has proposed its proactive action. However, each approach has a different reasoning mechanism and affects the future states in different ways. *HIRR* supports humans in reaching their intentions. It infers a human’s intention and suggests, or enacts, a sequence of actions to reach the human’s goal starting from the current state. *EqM* prevents the human from being in undesirable states by predicting possible state evolutions and reasoning on what is desirable and how available robot actions could create benefit.

Integrating the two systems is not trivial. Consider the hiking example in the opening of this article and suppose that in a given state *s*, *EqM* infers an opportunity to warn the human of hail, *Opp*_5_(*α*_warn_, *s*, 2). Suppose that at the same time, *HIRR* recognized that the human intention is to go hiking and infers to bring the compass to the human.

We have two competing goals for robot acting, and action selection needs to weigh them *via* a common scale. We propose a solution for integrating *EqM* and *HIRR* by turning the goal from *HIRR* into an opportunity of type *Opp*_0_, hence, *Opp*_0_(*α*_collect(compass)_, *s*, 0) and check its degree. In other words, we check the desirability of the states that would be achieved by the action when applied. Note that we use *Opp*_0_ here since the decisions by *HIRR* are meant to be acted upon immediately and do not use multiple-step look-ahead, just like *Opp*_0_. Once we have converted the individual outputs from *HIRR* and from *EqM* to a common format, that is, sets of opportunities, we collect all these opportunities into a pool, from which the action selection component (Figure 1) chooses an acting alternative.

To transform a *HIRR* acting decision into an opportunity of type *Opp*_0_, we temporarily modify the outcome of the *Des* function. In the *EqM* framework, the *Des* function does not take human intentions into account. It does not model states with unfulfilled human intentions as undesirable and those with fulfilled ones as desirable. Such a *Des* function would therefore not generate an opportunity corresponding to an unfulfilled intention. We therefore temporarily modify *Des* to decrease the desirability of the current state (Algorithm 3, line 3), modeling the undesirability of unfulfilled intention, and increase the desirability of the effects of an action that fulfills the intention (line 4): This allows the generation of an opportunity based on human intention recognition. For example, a state that would be desirable to the degree of 0.7 by itself might only be 0.1 desirable when a certain human intention has been recognized. In contrast, we increase the desirability of the effects that would manifest when an action of the human’s intention is applied, which would not be the case otherwise when no human intention was recognized, e.g., with recognized human intention *Des*(*α*(*s*)) = 0.9 and without recognized human intention *Des*(*α*(*s*)) = 0.3.

Algorithm 3 *HIRR* -Opp(*s*, *G*_*H*_)

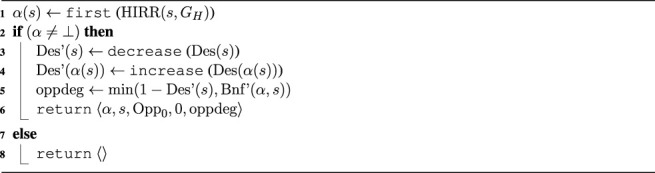

In our experiments, we have implemented the decrease and increase functions by scaling by a fixed value; exploring better ways to implement these steps is a matter for further investigation. The modified desirability function *Des*′(*s*) is used in line 8 to compute the degree of the opportunity of type *Opp*_0_ for applying action scheme *α* in the current state *s*. *α* is the first action in the recognized intention (action plan) returned by *HIRR* (line 1). Note that the computation in line 5 uses a modified *Bnf*′(*α*, *s*), which is based on *Des*′(*α*(*s*)). This opportunity and its degree are returned in line 6 and represent the opportunity based on human intention recognition.

Now that we have opportunities for acting based on prediction, as returned from Algorithm 2, and the one based on reasoning on human intention, as returned from Algorithm 3, we can decide which of them to enact in using the *action selection* Algorithm 4. This algorithm continuously checks if the state has changed (line 4), be it by changes in the environment or by the application of robot action. If so, it collects the opportunities coming from both proactivity systems, *EqM*(*s*, *K*) and *HIRR*(*s*, *G*_*H*_) (lines 5 and 6) and then choose one of these to be dispatched to the executive layer and enacted (line 8). The function Choose(), like the Choose() in Algorithm 2, can implement several strategies. In our experiments, Choose() selects the opportunity with the highest degree to be enacted. If there are several opportunities with the highest degree a decision is made by the opportunity type, how much benefit can be achieved and the size of the look-ahead. More discussion on these strategies can be found in the work of Grosinger et al., 2019.

Algorithm 4 Action Selection (*K*, *G*_*H*_)

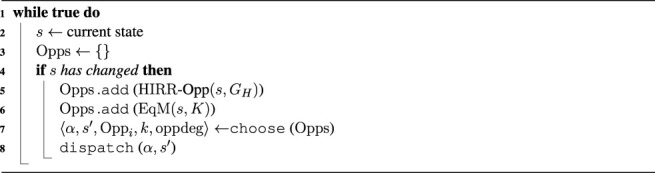

## 4 Illustrative experiments

In this section, we empirically illustrate the behavior of the presented approaches to proactive behavior by running the same simulated task with three different configurations of the system. We compare and analyze the outcomes of the system shown in Figure 1 when:

1. We only use intention-based proactivity (*HIRR*);
2. We only use predictive proactivity (*EqM*); and
3. We integrate both intention-based and predictive proactivity.

**FIGURE 2:**
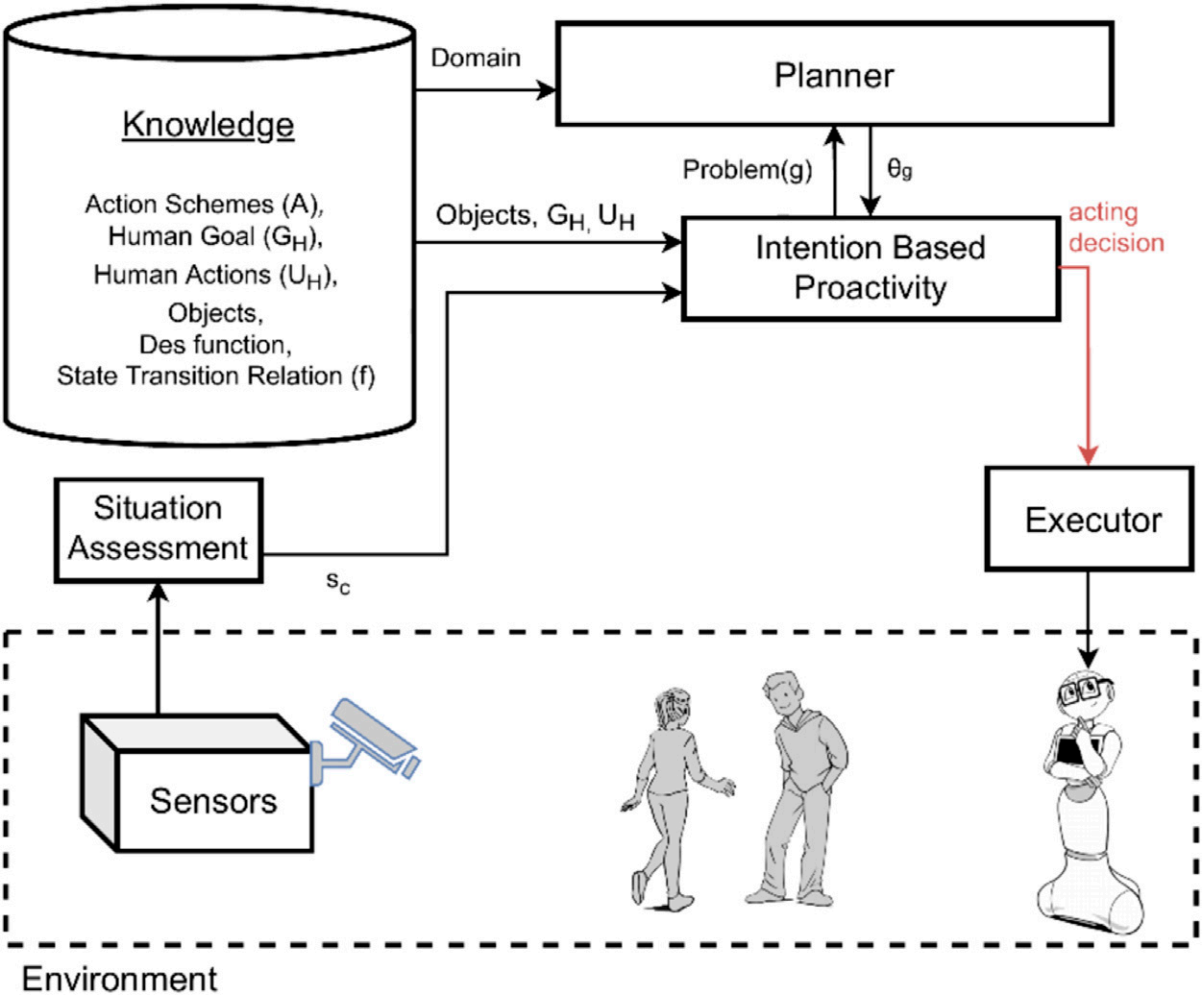
System model overview for human intention recognition and reasoning *HIRR*.

**FIGURE 3:**
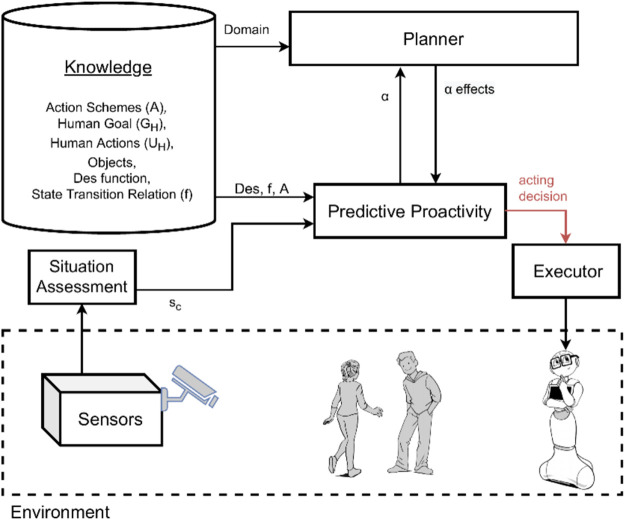
System model overview for equilibrium maintenance *EqM*.

Figure 2 and Figure 3 show the systems used for the first two experiments, while the integrated system used for the third experiment is the one previously shown in Figure 1. Note that these experiments are not meant to constitute a systematic empirical evaluation, but rather to provide a hands-on demonstration of how different results are obtained by applying the two types of proactivity discussed in the article, as well as by a combination of these two types, and to suggest that a combined approach might be needed.

The code is available in a “research bundle” on the European AI-on-demand platform, ai4europe.eu^4^. This research bundle includes the open source code, libraries, and a form of a notebook allowing users to interact with the framework by defining their environment.

### 4.1 Task description

**FIGURE 4:**
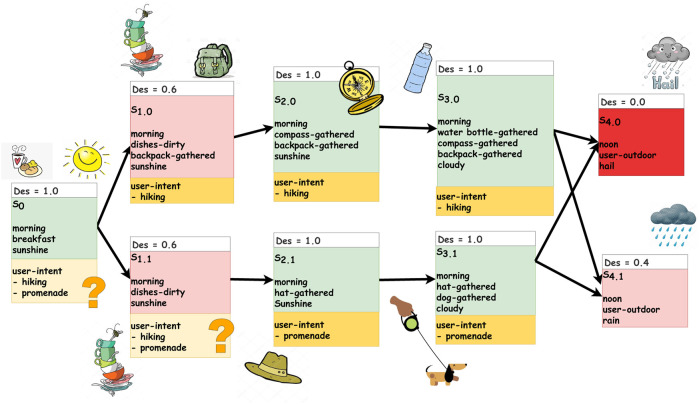
States and possible state transitions (free-run) in our scenario. The desirability values for each state are color coded, as well as indicated numerically. Green represents desirable states, while pink to red represents less desirable states. The more undesirable a state is, the more intense it’s red tone.

We define a hypothetical scenario where a human moves inside a house and collects objects to reach a goal. Figure 4 graphically represents the dynamic system Σ = ⟨*S*, *U*, *f*⟩ that models our scenario, where arrows show the state transitions *f* that correspond to possible evolutions of the environment (including actions by the human) if there is no interference from the robot. The figure also indicates the degree of desirability *Des*(*s*) of each state *s*. In addition to Σ and *Des*, the scenario includes the set of action schemes given in Table 1 and a set *G*_*H*_ of four human goals:

• **Hiking;** backpack collected, compass collected, water bottle collected, human is outside.
• **Promenade;** hat collected, dog collected, walking stick collected, human is outside.
• **Watch TV;** water bottle collected, sugar collected, tea collected, remote control collected.
• **Read Book;** glasses collected, book collected, tea collected, sugar collected.

**TABLE 1:** Actions that a human and a robot are capable to do. The name of the action (possibly including a parameter), preconditions of the actions, and the effects that will show after the action is applied, as well as who can do the action (human and/or robot), are provided.

Action	Precondition	Effect	Agent
Gather object	**And (**obj is not gathered, human at home**)**	Human gathered object	Human/robot
Leave object	**And (**obj is gathered, human at home**)**	Human not gathered object	Human/robot
Leave home	Human at home	Human not at home	Human
Suggest humans leave home	Human at home	Human not at home	Robot
Warn human	Human at home	**And (**human at home, human warned**)**	Robot
Clean dishes	**Or (**dishes dirty, dishes half dirty**)**	**Or (**dishes not dirty, dishes half dirty)	Human/robot

Each goal describes what must be true for it to be considered reached. For example, the goal of “hiking” is reached when it is true that a backpack, a compass, and a water bottle are collected and the human is outside. The actions to reach the individual goals can be done both by the human and the robot (except for going outside).

In our implementation, both the dynamic system and the action schemes are modeled in PDDL. Recall that PDDL includes a domain definition and a problem definition. In the domain definition, we model object definitions, predicate definitions for logical facts, and action definitions with preconditions and effects. In the problem definition, we model the initial state of the environment in a logical format, as well as the goal state. Actions are defined for *gathering* and *leaving objects*, for *telling the human they are ready to leave the house*, and for *cleaning the dishes*. The details are provided in Table 1. Some actions can be executed by the robot, some can be executed by the human, and some by both the human and the robot. The actions that are done by the human are observed by the robot, and based on them, the *HIRR* system recognizes the human’s intention. The *EqM* system, on the other hand, reasons about potential robot actions while taking into account the human’s actions which are part of the free-run (uncontrollable state transitions). Note that in this use case, all actions are deterministic except for *cleaning the dishes* which is nondeterministic: the action can have the effect that all dishes are clean or that they are still half dirty.

The defined robot actions are used in the *EqM* system to infer opportunities. *HIRR* recognizes human intention by inferring the human’s action plan. When the intention is recognized, *HIRR* can make the robot proactively carry out the rest of the human’s action plan on the human’s behalf. However, the human’s action plan toward their goal might contain an action that cannot be carried out by the robot. In that case, the robot transforms the action into a communication action where the robot tells the human what they should do. For example, after having collected all the necessary items, the human is supposed to leave the house to reach their goal of “hiking.” The robot can collect all necessary items but cannot leave the house, hence, it tells the human “Everything has been collected. You are ready to leave now for going hiking.”

The desirability function, *Des*, which computes the desirability degree of each state, is assumed given. In our example scenario, we consider one specific run where the state evolves as follows: *s*_0_, *s*_1.0_, *s*_2.0_, *s*_3.0_ (Figure 4). The system starts in *s*_0_ where the weather is nice, the time is morning, and the human is having breakfast. This state is very desirable, *Des*(*s*_0_) = 1.0. Later the state is changed to *s*_1.0_ where the weather is still nice and the time is still morning, but the human finished his or her breakfast so there are dirty dishes, and the human collected the backpack. This state is less desirable, *Des*(*s*_1.0_) = 0.6. Later the state evolves to *s*_2.0_, where the weather is cloudy and dishes are cleaned. In addition to the backpack, now the compass is collected. The last state chance is *s*_3.0_, where the weather is cloudy, the time is morning, and the human has collected the water bottle in addition to the previously collected belongings, backpack and compass. Note that the predicate “dishes-dirty” changes from true (in *s*_1.0_ and *s*_1.1_) to false (in *s*_2.0_ and *s*_2.1_). This is because the free-run state evolution models all uncontrollable state transitions, which include the environment and the human. Hence, the dishes not being dirty anymore means that the human has taken care of cleaning them.

### 4.2 Human intention recognition and reasoning only

We consider the scenario described in Figure 4 using only *HIRR* for achieving proactive agent activity. This means we evaluate the implementation of the method *HIRR* as described in Section 3.1. The architecture of the system is shown in Figure 2. Table 2 lists the recognized human intentions in the respective state and the proactive agent activity inferred.In *s*_0_, the *HIRR* cannot recognize yet what the human’s intention is, and it could be any of the four known human goals, going on a hike or going on a promenade, watching TV, or reading a book. Then the state advances to *s*_1.0_ where a backpack is collected. In this state, the *HIRR* is able to detect that the human’s intention is to go on a hike. The *HIRR* can infer to *proactively* bring the water bottle to the human as this is the next action inferred in the human’s action plan. The action is dispatched and the robot proactively brings the water bottle to the human (in simulation). Now the human has the backpack (gathered by the human him-/herself) and the water bottle (gathered by the robot). The state evolution advances to the next state $s_{2.0}^{\prime }$ . In this state also, a compass is gathered, which was done by the human. (For any state *s* in the free-run in Figure 4, *s*′ marks its equivalent on which robot action has been applied.) Again, the intent recognition detects that the human’s intention is going on a hike. The intention reasoning system detects that all necessary items for going on a hike have been collected. Therefore, it infers the *proactive* activity of notifying the human that he/she is ready to leave. Note that, in state $s_{2.0}^{\prime }$ (which is the state *s*_2.0_ plus applied robot action), the same predicates are true as in *s*_3.0_. This is eligible and expected as the robot’s proactive acting is doing part of the human’s action plan based on *HIRR*. Therefore, a state $s_{2.0}^{\prime }$ would not evolve into *s*_3.0_ (which is identical), but into states *s*_4.0_ or *s*_4.1_ where the human is outdoors. Note that, once the human has left the house, proactive interaction from our system with him/her is not possible. That is why there is no intention recognized in *s*_4.0_, *s*_4.1_. Note also that these states are quite undesirable (*Des*(*s*_4.0_) = 0.0 and *Des*(*s*_4.1_) = 0.4), as the user is outdoors while the weather conditions are unpleasant (rain) or even dangerous (hail). The algorithm for *HIRR* neither does any prediction of future states nor reasons about desirability/preference. Therefore, it is ignorant of the upcoming undesirable situation and cannot act on it.

**TABLE 2:** The state evolution and the proactive agent activity inferred in each state when using *HIRR* only.

State	Intention recognized	Proactive agent activity chosen—*HIRR*
*s*_0_	?	—
*s*_1.0_	Hiking	Gather water bottle
$s_{2.0}^{\prime }$	Hiking	Tell the human that he/she is ready to leave the house
*s*_3.0_	?	—

### 4.3 Equilibrium maintenance only

We again consider the use case described in Figure 4 but now using *EqM* only for achieving proactive agent activity. This means we test an implementation of the *EqM* algorithm as described in Section 3.2. Table 3 lists the opportunities for acting inferred in the respective state and the proactive agent activity to be enacted, i.e., the chosen opportunity. The outcome of *EqM* depends very much on the size of the prediction, *K*. In our system run, we set *K* = 2. (See Grosinger et al. (2019) for a discussion on the choice of the look-ahead horizon *K*.) We let *EqM* infer opportunities for acting that are current (*k* = 0), one-time step in the future (*k* = 1), and two-time steps in the future (*k* = 2).

**TABLE 3:** The state evolution and the proactive agent activity inferred in each state when using *EqM* only. Note that *α*_warn_ refers to warning the human of risk of bad/harmful weather conditions, *α*_clean_ refers to cleaning the dishes, i.e., putting them in the dishwasher, and *α*_gather(any)_ refers to gathering any object for the human.

State	Opportunities inferred	Proactive agent activity chosen
*s*_0_	*Opp*_3,4,5,6_(*α*_gather(any)_, *s*, 1) = 0.01	Clean dishes in 1 step
	*Opp*_3,4_(*α*_clean_, *s*, 1) = 0.4	
*s*_1.0_	*Opp*_0_(*α*_gather(any)_, *s*, 0) = 0.01	Clean dishes now
	*Opp*_0_(*α*_clean_, *s*, 0) = 0.4	
	*Opp*_1,2_(*α*_gather(any)_, *s*, 1) = 0.01	
	*Opp*_1,2_(*α*_gather(any)_, *s*, 2) = 0.01	
*s*_2.0_	*Opp*_5,6_(*α*_gather(any)_, *s*, 2) = 0.01	Warn for hail, effect seen in 2 steps
	*Opp*_5_(*α*_warn_, *s*, 2) = 1.0	
	*Opp*_6_(*α*_warn_, *s*, 2) = 0.6	
*s*_3.0_	*Opp*_5,6_(*α*_gather(any)_, *s*, 1) = 0.01	Warn for hail, effect seen in 1 step
	*Opp*_5_(*α*_warn_, *s*, 1) = 1.0	
	*Opp*_6_(*α*_warn_, *s*, 1) = 0.6	

The state evolution starts from state *s*_0_. The following opportunities are inferred: *EqM* does not infer to act in the current state, *s*_0_, because the human is having breakfast and the state is very desirable, *Des*(*s*_0_) = 1.0. However, when projecting the state one-time step into the future, *EqM* observes that the upcoming possible states will be less desirable, *Des*(*s*_1.0_) = 0.6 and *Des*(*s*_1.1_) = 0.6, because there will be dirty dishes from the breakfast. Therefore, *EqM* infers the opportunities for the robot to put the dishes in the dishwasher in the future, i.e., one step from now. There are no opportunities for acting in two-time steps. When the human gathers the backpack, state *s*_0_ evolves to *s*_1.0_. The state is not very desirable, *Des*(*s*_1.0_) = 0.6, because there are dirty dishes from the breakfast. *EqM* infers the opportunity for the robot to put the dirty dishes in the dishwasher now, in the current state *s*_1.0_. Note that, what before in *s*_0_ had been an opportunity for acting in the future is now an opportunity for acting in the present.When the human gathers the compass, the state evolves into *s*_2.0_. The state is desirable again, *Des*(*s*_2.0_) = 1.0, because the dishes are not dirty anymore. This means either the robot has enacted the opportunity of putting the dishes in the dishwasher in the previous state or putting the dishes in the dishwasher has happened through uncontrollable action as part of the free-run, i.e., the human has put the dishes in the dishwasher. In *s*_2.0_, there are no opportunities to consider the current state or take a look-ahead of *k* = 1. However, when *EqM* projects the state two steps into the future, it appears that the possible states are very undesirable, *Des*(*s*_4.0_) = 0.0, and quite undesirable, *Des*(*s*_4.1_) = 0.4. This is because the human will be outdoors and the weather will be very bad (rain) or even dangerous (hail). *EqM*, therefore, infers an opportunity for the robot to act now to prevent the future very undesirable outcome. More concretely, the robot *proactively* goes to the human and warns the human now to prevent him/her to be outdoors in the hail later. In case the warning is not heeded, in *s*_3.0_, the same opportunity for acting is inferred, only that now the look-ahead is a one-time step instead of two.

### 4.4 Human intention recognition and reasoning and equilibrium maintenance

As before, we consider the use case described in Figure 4 but now using both *HIRR* and *EqM* for achieving proactive agent activity. This means we have a system as described in Section 3.3. Table 4 lists the opportunities inferred by *HIRR* and the opportunities inferred by *EqM*, in the respective state, and marks which of them is chosen to be enacted. In *s*_0_, there are no opportunities by *HIRR* since the human intention cannot be unambiguously determined yet which yields zero opportunities for proactive acting (Section 3.1). *EqM*, on the other hand, does infer opportunities for acting in state *s*_0_. The one opportunity with the greatest degree, and hence, the one chosen, is an opportunity to clean the dishes in one-time step from now, *Opp*_3,4_(*α*_clean_, *s*_0_, 1).In *s*_1.0_, there is an opportunity coming from *HIRR* since the human intention is recognized as “hiking.” Hence, there is an opportunity to gather the water bottle, *Opp*_0_(*α*_gather(wb)_, *s*_1.0_, 0). The degree and the type of opportunity are computed according to Algorithm 3. Moreover, *EqM* produces opportunities in *s*_1.0_. The opportunity from *EqM* which has the highest degree is cleaning the dishes now—this is the opportunity from *s*_0_, now being of type *Opp*_0_ (to be applied now), while it was an opportunity for the future, *Opp*_3,4_, in *s*_0_. *HIRR*’s opportunity to gather the water bottle now is chosen to be enacted. How to choose between the opportunities from *HIRR* and *EqM* is determined by Algorithm 4.

**TABLE 4:** The state evolution and the proactive agent activity inferred in each state when using both *HIRR* and *EqM*. (Note that *α*_warn_ refers to warning the human of risk of bad/harmful weather conditions, *α*_clean_ refers to cleaning the dishes, i.e., putting the dishes in the dishwasher, *α*_gather(any)_ refers to gathering any object for the human, and *α*_leave_ refers to informing the human that he/she is ready to leave the house.)

State	Proactive acting—*HIRR*	Proactive acting—*EqM*	Chosen proactive action
*s*_0_		*Opp*_3,4,5,6_(*α*_gather(any)_, *s*, 1) = 0.01	Clean dishes in 1 step
		*Opp*_3,4_(*α*_clean_, *s*, 1) = 0.4	
*s*_1.0_	*Opp*_0_(*α*_gather(wb)_, *s*, 0) = 0.5	*Opp*_0_(*α*_gather(any)_, *s*, 0) = 0.01	Gather water bottle now
		*Opp*_0_(*α*_clean_, *s*, 0) = 0.4	
		*Opp*_1,2_(*α*_gather(any)_, *s*, 1) = 0.01	
		*Opp*_1,2_(*α*_gather(any)_, *s*, 2) = 0.01	
*s*_2.0_	*Opp*_0_(*α*_leave_, *s*, 0) = 0.5	*Opp*_5,6_(*α*_gather(any)_, *s*, 2) = 0.01	Warn for hail, effect seen in 2 steps
		*Opp*_5_(*α*_warn_, *s*, 2) = 1.0	
		*Opp*_6_(*α*_warn_, *s*, 2) = 0.6	
*s*_3.0_	*Opp*_0_(*α*_leave_, *s*, 0) = 0.5	*Opp*_5,6_(*α*_gather(any)_, *s*, 1) = 0.01	Warn for hail, effect seen in 1 step
		*Opp*_5_(*α*_warn_, *s*, 1) = 1.0	
		*Opp*_6_(*α*_warn_, *s*, 1) = 0.6	

The human now has the backpack (gathered by the human him-/herself) and the water bottle (gathered by the robot). The state evolution advances to the next state $s_{2.0}^{\prime }$ . Note that in state $s_{2.0}^{\prime }$ (which is the state *s*_2.0_ plus applied robot action), the same predicates are true as in *s*_3.0_ (Section 4.2). *HIRR* recognizes going on a hike as the intention of the human and proposes the opportunity to inform the human that he/she is ready to leave the house as all belongings for the hike have been packed. *EqM* proposes opportunities for warning the human about undesirable (rain) or possibly dangerous weather conditions (hail) as the human is predicted to be outdoors in the future, two-time steps from now. The opportunity coming from *EqM* (warning the human) has a higher degree, 0.6, than the opportunity coming from *HIRR* (“ready-to-go” message for the human) which has a degree of 0.5. Therefore, the combined system of *HIRR* and *EqM* chooses to dispatch the robot activity of warning the human.

In *s*_3.0_, since the conditions have not changed, *HIRR* infers the opportunity to confirm the human that they have gathered all necessary items and hence are ready to leave the house for going hiking. The warning of *EqM* has not been heeded in the previous state by the human, hence, *EqM* again infers to warn the human for the future unpleasant/dangerous weather, only now just one-time step into the future instead of two.

## 5 Discussion and conclusion

In this article, we have analyzed two approaches to proactivity: *HIRR*, which infers proactive actions by recognizing the human’s intended plans and taking over the next action in these plans, and *EqM*, which infers opportunities for acting by reasoning about possible future states and about what states are preferable. We have then defined a third approach that combines these two types of proactivity and have illustrated the three approaches in a sample use case. Our analysis shows that each approach can generate some proactive behaviors but not others. *HIRR* focuses on helping humans toward achieving their intentions, whereas *EqM* focuses on preventing humans to end up in undesirable situations. *EqM* does not consider humans’ intentions and therefore cannot generate proactive behavior to support the human to achieve them; *HIRR*, on the other hand, does not reason about how the state will evolve and about the overall desirability of future states and therefore cannot generate proactive behavior based on the predicted benefit of actions. The combined system can take into account both humans’ intentions and the desirability of future states.

A major aim of this article is to clarify that there are several ways to achieve proactive behavior and to show that a combined scheme may be needed. The next step will be to explore possible ways to achieve this combination. The admittedly naive way we proposed can be considered as a base case, which serves as an example. Many smarter and tighter forms of combination can be explored, and more work will be needed to do that. For instance, in our approach, *HIRR* and *EqM* independently propose proactive actions, and one of those is selected. This can be called *late integration*. Future work might investigate *early* forms of integration, where reasoning on human intentions, on available robot’s actions, on future states, and on preferences among those states is done in an integrated fashion. This tighter integration of *HIRR* and *EqM* will require a shared formulation for the two.

Our current framework is built on state descriptions that only consider the physical world. In future work, it will be interesting to include the inner world of humans. We plan to explore the use of techniques from the area of epistemic logic and epistemic planning to model the intentions, knowledge, and beliefs of human agents. The *Des* function and benefit of acting can then also take into account the preferences of mental states and how to bring preferable epistemic states about.

While we considered a single human in this article, both *HIRR* and *EqM* can in principle consider multiple humans: one would then have to track separately the single actions of each person and infer their intentions. If the humans are collaborating, *HIRR* could consider all of the humans’ actions together to infer the collective intention. *EqM* can fuse the single humans’ preferences in one overall *Des* function. Besides considering multiple humans, our system might also consider multiple proactive robots. New challenges may be posed about how to coordinate them but also opportunities may arise for solving tasks jointly.

The results of *EqM*, as well as the results of the combined system *HIRR* + *EqM*, strongly depend on the models of the dynamics of the system Σ, which determines the prediction of the state evolution (free-run), and on the modeling of preferences (*Des* function). To see this, consider the example in Section 4. In state *s*_1.0_, the opportunity of *HIRR*, to gather the water bottle, is chosen over the opportunity of *EqM*, to clean the dishes. If the desirability function modeled a stronger undesirability of dirty dishes, then the degree of the opportunity by *EqM* would have been higher and hence this opportunity would have been chosen to be enacted. Furthermore, consider a slightly modified state dynamics that differs from the one in Figure 4. In these modified state dynamics, the states at time 4 have weather conditions ‘sun’ and ‘clouds’ (instead of ‘rain’ and ‘hail’). In this use case, *EqM* would only infer to clean the dishes in state *s*_1.0_, but it would not support the human preparing for the hike because it is not reasoning on the human’s intention. *HIRR*, on the other hand, could provide a lot of support to the human to achieve his or her intention and would achieve a very desirable final outcome: the human out on a hike with all his or her belongings gathered.

In this article, we have simply assumed that both Σ and *Des* are given. Future work, however, might explore the use of machine learning to learn probabilistic models of state evolution. Moreover, hybrid techniques (model-based and data-driven) are conceivable which should take into account inferring the human’s intentions as this is an indicator of what the human will do next and thereby how the state will change. When eliciting and reasoning on preferences, ideally, the proactive agent should take into account the dynamic change of preferences, weigh personal against common, long-term against short-term preferences, and reason on the uncertainty of what is desirable.

Furthermore, the experiments reported in Section 4 are simple illustrative examples, which have been run using console inputs and outputs to replace physical sensing and actuation. Given the promising results, the next step will be to connect our system to a real Pepper robot (Pandey and Gelin, 2018), conduct a user study in a real domestic environment, and quantitatively evaluate the results. Since our system is based on general models, we also plan to test it on a variety of physical robot systems or interactive agents in diverse domains.

## Data Availability

The raw data supporting the conclusion of this article will be made available by the authors, without undue reservation.

## Footnotes

1
https://www.fast‐downward.org/

2 Inverse planning means working backward to infer the intention which caused the agent’s behavior (Baker et al., 2009). This is different from *backcasting* which is a method where the future desired conditions are envisioned and steps are then defined to attain those conditions (Holmberg and Robert, 2000).

3 This algorithm is a slightly modified version of the original version given by Grosinger et al. (2019); this is done to accommodate for the integration with *HIRR*.

4
https://www.ai4europe.eu/research/research-bundles/proactive-communication-social-robots
